# Duration of frontline therapy and impact on clinical outcomes in newly diagnosed multiple myeloma patients not receiving frontline stem cell transplant

**DOI:** 10.1002/cam4.5239

**Published:** 2022-09-24

**Authors:** Sikander Ailawadhi, Augustina Ogbonnaya, Sharanya Murty, Dasha Cherepanov, Bridgette Kanz Schroader, Dorothy Romanus, Eileen Farrelly, Ajai Chari

**Affiliations:** ^1^ Division of Hematology and Oncology Mayo Clinic Jacksonville Florida United States; ^2^ Xcenda, LLC Carrollton Texas United States; ^3^ Takeda Development Center Americas, Inc (TDCA) Lexington Massachusetts United States; ^4^ Hematology and Oncology Icahn School of Medicine at Mount Sinai New York New York United States

**Keywords:** chemotherapy, clinical management, multiple myeloma, target therapy

## Abstract

**Background:**

Extended first‐line therapy (1LT) has improved clinical outcomes in newly diagnosed multiple myeloma (NDMM). This retrospective study of NDMM patients evaluated the relationship between dose‐attenuation of 1LT and duration of therapy (DOT) and DOT on outcomes.

**Methods:**

Adults with NDMM not undergoing stem cell transplant (SCT) from January 1, 2012 toMarch 31, 2018 from the Integrated Oncology Network were included; 300 were randomly selected for chart review. 1LT DOT, time to next treatment (TTNT), progression‐free survival (PFS), and overall survival (OS) were estimated using Kaplan–Meier analysis. Marginal structural models evaluated relationships between DOT and TTNT, PFS, and OS at 2 years accounting for confounders and survival bias from the time‐dependent nature of DOT.

**Results:**

Of 300 chart‐reviewed patients, 93 were excluded for incomplete data or meeting exclusion criteria. Among 207 NDMM patients, median age was 74 years; 146 (70.5%) did not receive dose‐attenuation during 1LT. Patients with short DOT were older, frailer, with a higher comorbidity burden, and a significantly lower proportion had an Eastern Cooperative Oncology Group PS = 0. As DOT increased, more patients underwent dose‐attenuation (*p* < 0.0001). The median 1LT DOT was 20.9 (95% confidence interval [CI]: 13.9, 26.4) versus 4.2 months (95% CI: 3.2, 4.9) for patients receiving versus not receiving dose‐attenuation, respectively (*p* < 0.0001). After accounting for survival bias, confounder‐adjusted TTNT was prolonged with each additional month of 1LT (odds ratio [OR]: 0.76 [95% CI: 0.75, 0.78]); likelihoods of risks of disease progression (OR: 0.87 [95% CI: 0.86, 0.88]) and death at 2 years (OR: 0.72 [95% CI: 0.70, 0.74]) were reduced with each month of 1LT (*p* < 0.0001 for all outcomes).

**Conclusions:**

Dose‐attenuated 1LT was associated with longer DOT among patients with non‐SCT NDMM. Each additional month of 1LT was associated with a reduced adjusted likelihood of disease progression and death at 2 years. Dose‐attenuation of 1LT can extend DOT; longer DOT may improve clinical outcomes.

## Graphical abstract

Frequently, transplant‐ineligible patients are those who are frail and/or elderly, highlighting an unmet medical need for tolerable therapies; one approach to sustaining response balanced with tolerability is the use of maintenance or dose‐attenuated therapies. Among newly diagnosed multiple myeloma patients who did not receive stem cell transplant, dose‐attenuated first‐line therapy was associated with longer duration of therapy vs first‐line therapy without dose‐attenuation. Each additional month of first‐line therapy was associated with a reduced adjusted likelihood of disease progression and death at 2 years.

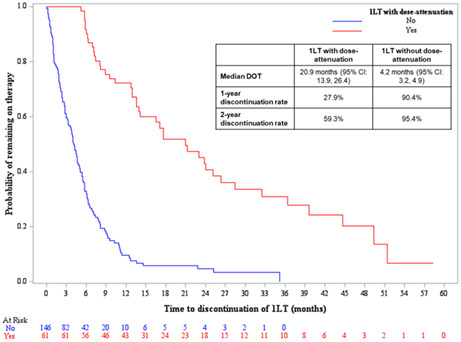

## 1 INTRODUCTION

Globally, multiple myeloma (MM) is the second most common hematologic malignancy and, despite treatment advances, remains an incurable disease.^1^ Because of this, the goals of therapy are to ensure the best possible response, delay disease progression, and prolong survival.^2^, ^3^, ^4^

First‐line therapy (1LT) should commence when a patient is diagnosed with active MM.^5^ Notably, subsequent classification of newly diagnosed multiple myeloma (NDMM) patients into hematopoietic stem cell transplant (SCT) eligible or ineligible is crucial in deciding which initial treatment is appropriate. Patients with SCT‐ineligible NDMM are generally older and/or have more comorbid conditions than patients who are SCT candidates; this ultimately makes the choice of frontline treatment in SCT‐ineligible patients more complex.^6^, ^7^, ^8^

Initial treatment options for patients with non‐SCT NDMM are based on data and guidelines indicating that continued treatment to progression is associated with improved outcomes, regardless of depth of response, compared to fixed‐duration therapy.^9^, ^10^, ^11^ Extending treatment in frail populations that cannot pursue intensive consolidation therapy in an attempt to extend or deepen response remains important as subsequent lines of therapy exhibit decreasing time to progression.^12^

Despite the clinical benefits associated with treatment to progression, real‐world analyses in the non‐SCT NDMM patient population have demonstrated worse outcomes than those observed in clinical trials reflecting the impact of non‐selected patient populations and patient or physician willingness to treat to progression.^13^ Additionally, these analyses have shown that duration of 1LT is shorter than the time to next treatment (TTNT) initiation suggesting intolerance to continued therapy.^12^, ^13^, ^14^ In an attempt to improve outcomes, various treatment options to extend the duration of 1LT have been employed, including administration of lenalidomide or lenalidomide/dexamethasone (Rd) maintenance following induction.^15^, ^16^ Improvements in progression‐free survival (PFS) for patients with non‐SCT NDMM were observed with both treatment approaches.

Overall, the published data suggest that prolonging therapy by dose or schedule adjustments, such as Rd followed by lenalidomide maintenance (Rd‐R) versus continuous Rd, in elderly NDMM patient populations may result in improved event‐free survival.^17^ Elderly patients with NDMM receiving Rd‐R versus continuous Rd had a longer median event‐free survival (10.4 months vs. 6.9 months; hazard ratio [HR]: 0.70; 95% confidence interval [CI]: 0.51, 0.95; *p* = 0.02) but similar median PFS (20.2 months vs. 18.3 months; HR: 0.78 [95% CI: 0.55, 1.10]; *p* = 0.16) and 3‐year OS (74% vs. 63%; HR: 0.62 [95% CI: 0.37, 1.03]; *p* = 0.06).^17^ Most published data on maintenance or continuous therapy in patients with SCT‐ineligible NDMM have been reported in controlled clinical trials which limits the generalizability of the results to real‐world patients who do not meet trial eligibility criteria.^18^, ^19^

Current practice guidelines for the frontline treatment of patients with SCT‐ineligible NDMM recommend maintenance therapy in patients responding to primary (i.e., induction) therapy.^11^ Consequently, evaluations of outcomes with maintenance therapy in 1LT are of increasing importance for healthcare providers and other stakeholders. Considering this evolving MM treatment landscape, we examined the use of maintenance therapy (“dose‐attenuated” therapy) and outcomes in real‐world patients with non‐SCT NDMM. Specifically, we assessed whether dose‐attenuation of 1LT is associated with a longer duration of therapy (DOT) and the impact of longer first‐line DOT on treatment outcomes, utilizing marginal structural models (MSMs), which account for survival bias and changing patient and disease characteristics over time, thus controlling for the higher propensity of patients with more favorable characteristics and/or more indolent disease to stay on treatment.

## 2 METHODS

### 2.1 Study design and data source

This was a retrospective analysis of a random sample of patients with NDMM initiating 1LT from an electronic healthcare record (EHR) database, the Integrated Oncology Network (ION) database, with data supplemented further with medical chart review data. Treatment data obtained from the chart review was verified independently by an expert MM oncologist. The ION EHR contains disease‐ and treatment‐specific variables from 350 unique providers from over 25 geographically diverse, large, community‐based oncology practices encompassing more than 650,000 patients. The data are certified as de‐identified in line with the US Health Insurance Portability and Accountability Act statistical de‐identification rules. Institutional review board (IRB) approval for this study was obtained from the Advarra IRB.

The study period was January 1, 2012 through March 31, 2018. Treated MM patients were initially identified during the enrollment period from July 1, 2012 through December 31, 2017. The index diagnosis date was defined as the first chronologically occurring MM diagnosis during the enrollment period. Subsequently, the first chronologically occurring date of an MM chemotherapy during the enrollment period was defined as the index treatment date. The baseline period was the 6‐month period prior to index treatment date and was used to characterize the patients. The follow‐up period (including the index treatment date) was variable for all patients; patients were followed longitudinally until death, loss to follow‐up (defined as no additional data available prior to the end of study period), or end of study period (March 31, 2018). See Figure SS1.

### 2.2 Patient identification criteria

The population of interest in this study were patients with NDMM who did not undergo SCT as part of 1LT. Patients were included in the study if they had a MM diagnosis between July 1, 2007 through March 31, 2018; received MM‐directed chemotherapy following the index diagnosis date during the enrollment period; and were aged ≥18 years on the index diagnosis date. Patients were excluded if they had evidence of any anti‐cancer systemic therapy or SCT prior to index diagnosis date (except for dexamethasone given for <90 days), no evidence of MM‐directed chemotherapy, missing gender or date of birth, or if they did not have activity in the EHR after index diagnosis date.

**FIGURE 1:**
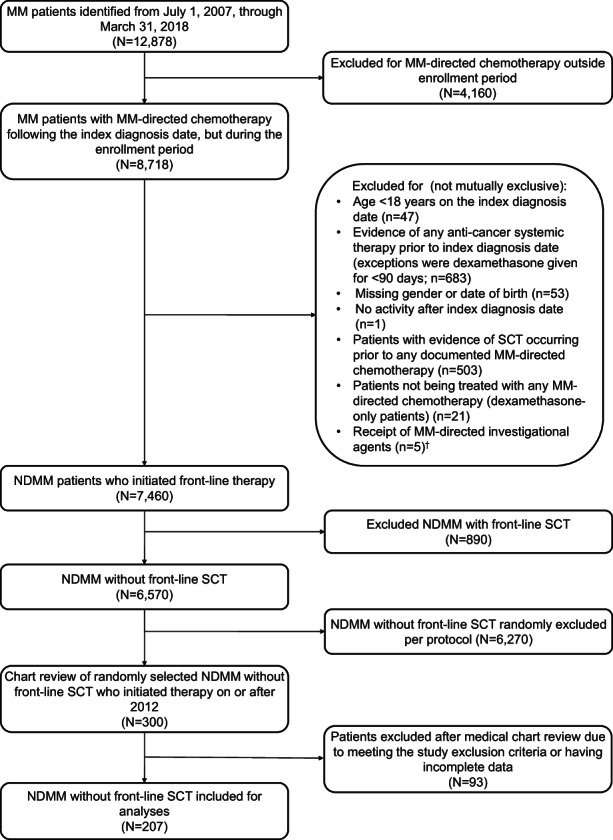
Identification of patients with NDMM without front‐line SCT. ^†^ Receipt of MM‐directed investigational agents included, for example, patients receiving ixazomib, daratumumab, or elotuzumab the LOT start date was prior to 2015 (year of initial Food and Drug Administration approval). LOT, line of therapy; MM, multiple myeloma; NDMM, newly diagnosed multiple myeloma; SCT, stem cell transplant.

Of the patients who met the study selection criteria, a random sample of 300 patients were selected from the pool of patients who initiated 1LT after July 1, 2012, and manual chart review was performed for these patients to provide a clinically enriched dataset. Randomly selecting patients from this time period provided the most contemporary analysis of treatment patterns and outcomes available in the data set. Additional details provided in Figure 1.

### 2.3 Study variables

#### 2.3.1 Treatment patterns

1LT was defined as induction therapy with or without subsequent dose‐attenuated therapy. Induction therapy and dose‐attenuated therapy were captured through abstraction of the treating physician's medical record documentation and confirmed by an expert MM oncologist based on conceptual definitions below.

Dose‐attenuated therapy was conceptually defined with input from expert MM oncologists as any dose or regimen adjustment intended to sustain response to induction therapy in the absence of progression, relapse, or toxicity. Accordingly, changes from a triplet to a doublet‐ or mono‐therapy, or doublet‐therapy accompanied by a dose reduction and/or a sustained decrease in frequency of administration—from an induction regimen to a subset of the initial regimen, or to a single agent—were deemed to constitute dose‐attenuated therapy. Conversely, a decrease of frequency/dose followed by resumption of the original frequency/dose was not considered dose‐attenuation (to account for real‐world practice when patients may not be seen exactly on schedule or may experience transient toxicity).

A new line of therapy (LOT) was determined based on EHR data and input from MM oncologists, when a planned therapy was modified to include other treatments per NCCN Clinical Practice Guidelines in Oncology (NCCN Guidelines®) (alone or in combination; for example, switch, including within the same mechanism of action, or add‐on of an agent, other than steroids) as a result of progressive disease, relapse, sub‐optimal response/lack of response, or toxicity.^11^, ^20^, ^21^ See the Appendix S1 for the conceptual definition used by the MM oncologists.

1LT characteristics included the induction therapy (all regimens, by drug category [proteasome inhibitor (PI)‐based, PI/immunomodulatory drug (IMID)‐based, IMID‐based, alkylator‐based, PI/alkylator‐based, IMID/alkylator‐based, PI/IMID/alkylator‐based], and by regimen drug count [≥3 drugs, ≤2‐drugs]), dose‐attenuated therapy regimen, reason for 1LT discontinuation for those not receiving dose‐attenuated therapy, and reason for discontinuation of dose‐attenuated therapy for those who received dose‐attenuated therapy. The opportunity to utilize dose‐attenuation was evaluated in patients not receiving dose‐attenuated therapy by assessing initiation of 2LT within 60 days of discontinuing 1LT. See the Appendix S1 for details.

#### 2.3.2 Baseline clinical characteristics

Baseline characteristics included demographics, year of MM diagnosis, year of treatment initiation, International Staging System (ISS) stage, immunoglobulin class, cytogenetic risk level, Eastern Cooperative Oncology Group (ECOG) performance status (PS), modified frailty index (based on Charlson Comorbidity Index [CCI] and age),^8^ CCI^22^; comorbidities (diabetes, thromboembolic disease, peripheral neuropathy, cardiovascular disease, prior non‐MM cancer); calcium elevation (hypercalcemia), renal insufficiency, anemia, bone disease (CRAB) symptoms^23^, ^24^; time from diagnosis to 1LT, and length of follow‐up (Table 1; Appendix S1).

**TABLE 1:** Demographic and clinical baseline characteristics in patients with NDMM without front‐line SCT

Characteristics	All Patients	Duration of 1LT				*p*‐value*
		0–3 months	3–6 months	6–12 months	12+ months	
	*N* = 207	*n* = 64	*n* = 45	*n* = 45	*n* = 53	
Age in years (mean, SD)	73.9 (10.9)	74 (12.2)	74.6 (9.7)	74 (11.3)	73 (9.9)	0.7465
Age group (*n*, %)						
≤64	36 (17.4)	11 (17.2)	7 (15.6)	10 (22.2)	8 (15.1)	0.6917
65–74	57 (27.5)	14 (21.9)	13 (28.9)	11 (24.4)	19 (35.8)	
75+	114 (55.1)	39 (60.9)	25 (55.6)	24 (53.3)	26 (49.1)	
Male (*n*, %)	123 (59.4)	38 (59.4)	25 (55.6)	25 (55.6)	35 (66)	0.6776
Race (*n*, %)						
African American	34 (16.4)	5 (7.8)	9 (20)	10 (22.2)	10 (18.9)	0.4759
Caucasian	115 (55.6)	38 (59.4)	24 (53.3)	23 (51.1)	30 (56.6)	
Unknown/Other	58 (28)	21 (32.8)	12 (26.7)	12 (26.7)	13 (24.5)	
Geographic region (*n*, %)						
Midwest	12 (5.8)	7 (10.9)	3 (6.7)	1 (2.2)	1 (1.9)	0.2431
Northeast	0 (0)	0 (0)	0 (0)	0 (0)	0 (0)	
South	183 (88.4)	52 (81.3)	39 (86.7)	41 (91.1)	51 (96.2)	
West	12 (5.8)	5 (7.8)	3 (6.7)	3 (6.7)	1 (1.9)	
Treatment index year (*n*, %)						
2012	1 (0.5)	0 (0)	1 (2.2)	0 (0)	0 (0)	0.5166
2013	46 (22.2)	13 (20.3)	11 (24.4)	9 (20)	13 (24.5)	
2014	59 (28.5)	16 (25)	14 (31.1)	10 (22.2)	19 (35.8)	
2015	39 (18.8)	13 (20.3)	7 (15.6)	8 (17.8)	11 (20.8)	
2016	44 (21.3)	14 (21.9)	11 (24.4)	13 (28.9)	6 (11.3)	
2017	18 (8.7)	8 (12.5)	1 (2.2)	5 (11.1)	4 (7.5)	
Duration of time from index diagnosis date to 1LT, months^a^						
Median (IQR)	0.6 (0.9)	0.5 (0.7)	0.8 (1.0)	0.7 (1.1)	0.5 (0.5)	0.0695
Duration of time from 1LT to end of follow‐up, months^a^						
Median (IQR)	14.2 (24.5)	2.6 (12.5)	13.8 (17.6)	13.7 (13.3)	30.9 (23.3)	<0.0001*
ECOG PS (*n*, %)						
0	29 (14)	6 (9.4)	6 (13.3)	4 (8.9)	13 (24.5)	0.0376*
1	69 (33.3)	20 (31.3)	15 (33.3)	19 (42.2)	15 (28.3)	
2+	48 (23.2)	20 (31.3)	8 (17.8)	14 (31.1)	6 (11.3)	
Unknown	61 (29.5)	18 (28.1)	16 (35.6)	8 (17.8)	19 (35.8)	
ISS Stage (*n*, %)						
Stage 1	22 (10.6)	9 (14.1)	3 (6.7)	3 (6.7)	7 (13.2)	0.6087
Stage 2	30 (14.5)	6 (9.4)	10 (22.2)	6 (13.3)	8 (15.1)	
Stage 3	60 (29)	21 (32.8)	13 (28.9)	11 (24.4)	15 (28.3)	
Unknown	95 (45.9)	28 (43.8)	19 (42.2)	25 (55.6)	23 (43.4)	
Immunoglobulin class (*n*, %)						
IgA	38 (18.4)	9 (14.1)	14 (31.1)	5 (11.1)	10 (18.9)	0.1614
IgD	3 (1.4)	1 (1.6)	1 (2.2)	0 (0)	1 (1.9)	
IgE	0 (0)	0 (0)	0 (0)	0 (0)	0 (0)	
IgG	91 (44)	23 (35.9)	16 (35.6)	22 (48.9)	30 (56.6)	
IgM	0 (0)	0 (0)	0 (0)	0 (0)	0 (0)	
Light chain only	51 (24.6)	20 (31.3)	9 (20)	13 (28.9)	9 (17)	
Biclonal	0 (0)	0 (0)	0 (0)	0 (0)	0 (0)	
Other	1 (0.5)	1 (1.6)	0 (0)	0 (0)	0 (0)	
Unknown	23 (11.1)	10 (15.6)	5 (11.1)	5 (11.1)	3 (5.7)	
Cytogenetic risk (*n*, %)						
Normal	81 (39.1)	23 (35.9)	17 (37.8)	16 (35.6)	25 (47.2)	0.7301
High risk	39 (18.8)	14 (21.9)	6 (13.3)	10 (22.2)	9 (17)	
Unknown	87 (42)	27 (42.2)	22 (48.9)	19 (42.2)	19 (35.8)	
Quan CCI (mean, SD)	2.43 (2.28)	2.38 (1.95)	2.56 (2.42)	2.73 (2.86)	2.11 (2)	0.8894
Quan CCI category (*n*, %)						
0	56 (27.1)	12 (18.8)	13 (28.9)	12 (26.7)	19 (35.8)	0.3678
1	15 (7.2)	6 (9.4)	2 (4.4)	5 (11.1)	2 (3.8)	
2+	136 (65.7)	46 (71.9)	30 (66.7)	28 (62.2)	32 (60.4)	
Select co‐morbid conditions (*n*, %)						
Diabetes	46 (22.2)	13 (20.3)	9 (20)	13 (28.9)	11 (20.8)	0.6853
Thromboembolic disease	4 (1.9)	0 (0)	0 (0)	4 (8.9)	0 (0)	0.0040*
Peripheral Neuropathy	12 (5.8)	1 (1.6)	3 (6.7)	6 (13.3)	2 (3.8)	0.0706
Cardiovascular Disease	20 (9.7)	6 (9.4)	5 (11.1)	5 (11.1)	4 (7.5)	0.9331
Prior non‐MM cancer	11 (5.3)	5 (7.8)	3 (6.7)	2 (4.4)	1 (1.9)	0.5080
Modified frailty index (*n*, %)^b^						
0 (fit)	36 (17.4)	7 (10.9)	8 (17.8)	9 (20.0)	12 (22.6)	0.6909
1 (intermediate)	73 (35.3)	22 (34.4)	17 (37.8)	15 (33.3)	19 (35.8)	
2+ (frail)	98 (47.3)	35 (54.7)	20 (44.4)	21 (46.7)	22 (41.5)	
CRAB comorbidities (*n*, %)						
Renal Insufficiency	138 (66.7)	48 (75)	26 (57.8)	32 (71.1)	32 (60.4)	0.1760
Anemia	191 (92.3)	57 (89.1)	43 (95.6)	42 (93.3)	49 (92.5)	0.6914
Hypercalcemia	46 (22.2)	18 (28.1)	9 (20)	10 (22.2)	9 (17)	0.5200
Bone disease	103 (49.8)	33 (51.6)	25 (55.6)	22 (48.9)	23 (43.4)	0.6685
1 L induction regimen drug class categories (*n*, %)						
IMID‐based	41 (19.7)	13 (20.3)	8 (17.8)	9 (20.0)	11 (20.8)	0.6962
PI‐based	59 (28.5)	18 (28.1)	17 (37.8)	13 (28.9)	11 (20.8)	
Alkylator‐based	1 (0.5)	0 (0)	1 (2.2)	0 (0)	0 (0)	
PI/IMID‐based	63 (30.4)	17 (26.6)	11 (24.4)	16 (35.6)	19 (35.8)	
PI/alkylator‐based	39 (18.8)	16 (25.0)	7 (15.6)	6 (13.3)	10 (18.9)	
IMID/alkylator‐based	1 (0.5)	0 (0)	0 (0)	0 (0)	1 (1.9)	
PI/IMID/alkylator‐based	2 (1.0)	0 (0)	1 (2.2)	0 (0)	1 (1.9)	
Other	1 (0.5)	0 (0)	0 (0)	1 (2.2)	0 (0)	

Abbreviations: 1LT, first‐line therapy; CCI, Charlson comorbidity index; CRAB, calcium elevation (hypercalcemia), renal insufficiency, anemia, bone disease; ECOG PS, Eastern Cooperative Oncology Group Performance Status; IMID, immunomodulatory drug; ISS, International Staging System; MM, multiple myeloma; n/a, not applicable; PI, proteosome inhibitor; SD, standard deviation.

* Statistically significant results. Chi‐square test was used for categorical variables. Fisher exact test was used for binary variables when the expected cell count was less than 5. Monte Carlo exact was used for categorical variables when the expected cell count was less than 5. ANOVA was used for continuous variables.

^a^ Not Kaplan–Meier estimates.

^b^ Based on CCI score and age.

### 2.4 Outcome measures

First‐line DOT was defined as the time from the start of the 1LT induction regimen to the end of all drug components of the 1LT regimen, including dose‐attenuation (if observed). A regimen that ended because of discontinuation or death (event) was not censored. A regimen that ended because of the end of study/follow‐up, was considered incomplete; and therefore, these observations were censored at the date of last follow‐up. Gaps between drug administrations were not assessed, and end of DOT was defined based on treatment discontinuation date; if a discontinuation date was not noted, it was assumed that the patient was still on therapy unless they died.

TTNT, a proxy for PFS, was defined as the time from the start of 1LT to the start of 2LT or death (whichever event occurred earlier). Patients were censored at the date of last follow‐up if they did not initiate 2LT, died within the follow‐up period, or were lost to follow‐up.

PFS was defined as the time from the start of 1LT to the earliest of the date of progression (event) or death (event) before the start of 2LT; dates of progression events were abstracted from patients' medical charts based on documentation by treating physicians, (described above). Patients who did not experience disease progression or death before the start of 2LT were censored at the beginning of 2LT (if they initiated 2LT) or at the end of follow‐up (if they did not initiate 2LT), whichever occurred first.

OS was defined as the time from the start of 1LT to the date of death from any cause for patients who died. Observations for patients who were alive until study end date or were lost to follow‐up were censored on the date of last follow‐up/study end date (whichever occurred first).

### 2.5 Statistical analysis

Descriptive analyses were performed to examine demographics, baseline clinical characteristics, induction treatment patterns, and dose‐attenuated treatment patterns in the overall population and stratified by DOT time intervals (≤3 months, >3 and ≤6 months, >6 and ≤ 12 months, and > 12 months [derived from observed treatment duration patterns]). Univariate comparisons between the DOT groups were conducted using Chi‐square tests for categorical variables, Fisher's exact tests for binary variables when expected counts were <5 observations, Monte Carlo exact tests for categorical variables when expected counts were <5 observations and ANOVA for continuous variables.

Kaplan–Meier analysis was used to examine DOT among all patients and by receipt of dose‐attenuated 1LT. Statistical comparison was done using the log‐rank test. TTNT, PFS, and OS were described for the overall population. Median TTNT, PFS, and OS and rates at 1 and 2 years were estimated using Kaplan–Meier analyses.

Marginal structural models (MSM)^25^ were used to evaluate the impact of DOT on TTNT, PFS, and OS to account for the time‐dependent nature of DOT and time‐varying confounders. First, the probability of continuing 1LT at each month from initiation of 1LT was estimated using a logistic regression model with being on treatment at each month as the dependent variable. Second, the probability of staying in the study at each month from the initiation of 1LT was estimated also using logistic regression, with being in the study at each month as the dependent variable. In both logistic regression models, covariates that remained constant were age, gender, race, region, year of index treatment start, ISS stage, immunoglobulin class, cytogenetic risk, frailty, CCI, and time from diagnosis to initiation of 1LT, while the time‐varying covariates that were updated monthly included ECOG PS, diabetes, thromboembolic disease, peripheral neuropathy, non‐MM cancer, and cardiovascular disease. The propensity scores derived from the logistic regression models were used to generate weights at each month, by multiplying the probability of being on 1LT at each month and the probability of being in the study at each month. Indicators (yes vs. no) for the outcomes (TTNT, PFS, and OS) at each month were defined based on whether or not the outcome has occurred in each month. These probabilities reflect the extent to which observations with certain characteristics are under‐represented or over‐represented in the sample with the respect to a target population in which these characteristics are balanced across DOT groups. The outcome indicators were modeled using logistic regression including the weights and the covariates to determine the average‐odds of having the outcome of interest for one additional month since the time of initiation of 1LT at 2 years of 1LT. The outcomes (TTNT/PFS/OS) were truncated at 2 years, given extensive censoring and a small number of patients remaining following 2 years of follow‐up (*n* = 33, 27, and 62 for TTNT, PFS, and OS, respectively). Results from the MSM were summarized as multivariable adjusted average ORs with associated 95% CIs, indicating the average adjusted odds of having a given outcome with each additional month of continuous 1LT among those who continued versus the average adjusted odds among those who discontinued 1LT.

All analyses were performed using SAS® version 9.4 (SAS Institute).

## 3 RESULTS

### 3.1 Baseline characteristics

A total of 207 patients were eligible for inclusion in the study following chart abstraction (Figure 1). Patients were categorized by DOT of 0–3 months (*n* = 64), 3–6 months (*n* = 45), 6–12 months (*n* = 45), and 12+ months (*n* = 53). Overall, patient characteristics were similar across the DOT categories with few exceptions, ECOG PS, CI score, modified frailty index score, and thromboembolic disease (Table 1). Over half of the patients were 75 years of age or older (55.1%), and across all DOT groups, the 0–3 DOT had the highest proportion of patients in this age group and the 12+ DOT group had the lowest proportion of patients in this age group. The majority of patients were male (59.4%) and Caucasian (55.6%). In terms of comorbidities, 65.7% of patients had a CCI score ≥2, with the 0–3 DOT group having the highest proportion of patients with CCI ≥2 and the 12+ DOT group having the lowest proportion of patients with CCI ≥2 across the DOT groups. The modified frailty index classified 47.3% of patients as being frail, and the proportion of frail patients was highest in the 0–3 DOT group and lowest in the 12+ DOT group. ECOG PS was ≥2 for 23.2% of patients (29.5%, unknown); patients with 12+ months of DOT tended to have lower ECOG PS with 11.3% of patients having an ECOG PS of ≥2 (35.8%, unknown) relative to those with DOT <12 months. ISS stage 3 was reported in 29.0% of patients (45.9%, unknown) and the most common immunoglobulin classes were IgG (44%), light chain (24.6%), and IgA (18.4%). High‐risk cytogenetics were reported in 18.8% of patients (42%, unknown). All patients had ≥1 CRAB symptom, with 92.3%, 66.7%, 49.8%, and 22.4% having anemia, renal insufficiency, bone disease, and hypercalcemia, respectively. Diabetes was the most common other comorbid condition (22.2%).

The median (interquartile range [IQR]) time from MM diagnosis to initiation of 1LT was 0.6 (0.9) months, and median (IQR) follow‐up time from treatment initiation was 14.2 (24.5) months.

### 3.2 Treatment patterns

**FIGURE 2:**
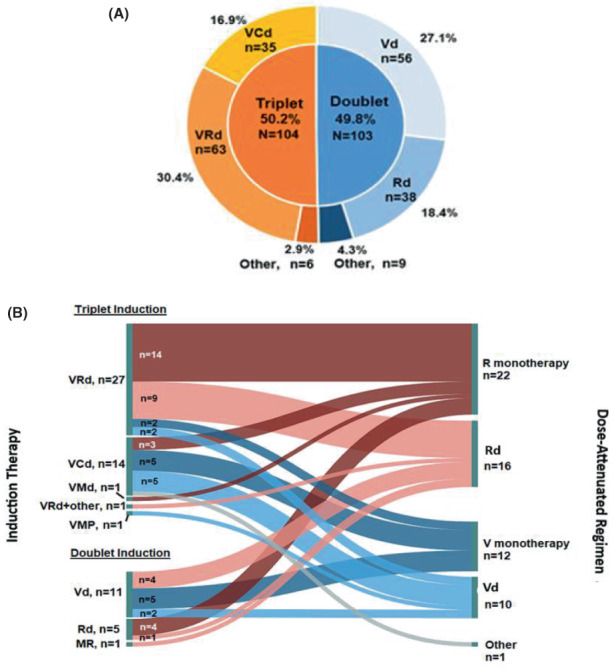
Induction (A) and dose‐attenuated regimens (B) in 1LT. (A) Induction therapy in patients with NDMM without front‐line SCT (*N* = 207). ^†^ One patient in the VCd group received prednisone instead of dexamethasone. ^‡^ Other included VMP (*n* = 2) and VMd (*n* = 1); Vd + vincristine (*n* = 1), and VCd + lenalidomide±etoposide and vincristine (*n* = 2). ^§^ Other included bortezomib ±cyclophosphamide (*n* = 3); lenalidomide ±melphalan (*n* = 3); dexamethasone ± thalidomide (*n* = 2); and melphalan + prednisone (*n* = 1). (B) Dose‐attenuated therapy after induction therapy in patients with NDMM without front‐line SCT (*n* = 61). ^†^ Other included cyclophosphamide + dexamethasone.1LT, first‐line therapy; C, cyclophosphamide; d, dexamethasone; M, melphalan; P, prednisone; R, lenalidomide; V, bortezomib.

Figures 2A and 2B present induction and dose‐attenuated regimens in 1LT. Induction therapy consisted of 3‐drug regimens or greater in 50.2% and 2‐drug regimens or fewer in 49.8% of patients. The most common induction 1LT regimens were bortezomib, lenalidomide, dexamethasone (VRd, 30.4%), Vd (27.1%), Rd (18.4%), and bortezomib, cyclophosphamide, and dexamethasone (VCd, 16.4%). Correspondingly, the most common drug categories were PI/IMID‐based (30.4%), PI‐based (28.5%), IMID‐based (19.8%), and PI/alkylator‐based (18.8%) regimens. Reasons for discontinuing treatment with VRd, Vd, Rd, and VCd are provided in Table 2.

**TABLE 2:** Reasons for discontinuing the top four first‐line induction regimens

Discontinuation reasons, *n* (%)	Top four first‐line induction regimens in 207 patients with NDMM without front‐line SCT			
	VRd (*N* = 63)	Vd (*N* = 56)	Rd (*N* = 38)	VCd (*N* = 34)
Adverse event/toxicity	8 (12.7)	2 (3.6)	8 (21.1)	3 (8.8)
Death	9 (14.3)	7 (12.5)	8 (21.1)	4 (11.8)
Disease progression	7 (11.1)	11 (19.6)	6 (15.8)	2 (5.9)
Going to maintenance	4 (6.3)	1 (1.8)	2 (5.3)	1 (2.9)
Lack of response	2 (3.2)	1 (1.8)	0 (0)	1 (2.9)
Maximum response to therapy achieved	16 (25.4)	11 (19.6)	1 (2.6)	14 (41.2)
Patient/family preference	2 (3.2)	2 (3.6)	0 (0)	2 (5.9)
Physician preference	0 (0)	1 (1.8)	1 (2.6)	0 (0)
Planned therapy end	4 (6.3)	2 (3.6)	0 (0)	1 (2.9)
Other	6 (9.5)	5 (8.9)	3 (7.9)	1 (2.9)
Unknown	3 (4.8)	10 (17.9)	2 (5.3)	1 (2.9)
Treatment ongoing	2 (3.2)	3 (5.4)	7 (18.4)	4 (11.8)

Abbreviations: NDMM, newly diagnosed multiple myeloma; Rd, lenalidomide, dexamethasone; SCT, stem cell transplant; VCd, bortezomib, cyclophosphamide, dexamethasone; Vd, bortezomib, dexamethasone; VRd, bortezomib, lenalidomide, dexamethasone.

The majority of patients did not undergo dose‐attenuation during 1LT (*n* = 146, 70.5%). However, as first‐line DOT increased, the number of patients receiving dose‐attenuation increased (*p* < 0.0001).

Among patients who did not receive dose‐attenuated therapy during 1LT (*n* = 146), 87.7% (*n* = 128) discontinued induction therapy; the most common reasons for discontinuation were death (22.7%), progression of disease (21.1%), and adverse event (AE)/toxicity (16.4%). The majority of patients who discontinued induction therapy had ≥60 days of follow‐up after discontinuation (59.4%). Of those who discontinued 1LT induction without dose‐attenuation, 69.9% of patients did not initiate 2LT within 60 days of discontinuing 1LT; this may indicate patients who discontinued 1LT (without dose‐attenuation) did not rapidly progress, suggesting that these patients had the opportunity to receive dose‐attenuation.

Among patients who received dose‐attenuated therapy (*n* = 61, 29.5%), the most commonly administered dose‐attenuated 1LT regimens were lenalidomide (36.1%), Rd (26.2%), bortezomib (19.7%), and Vd (16.4%). Among these patients, 68.9% (*n* = 42) discontinued the dose‐attenuated therapy during the study period. The most common reasons for discontinuation were progression of disease (*n* = 10, 23.8%), AE/toxicity (*n* = 9, 21.4%), and death (*n* = 7, 16.7%).

### 3.3 First‐Line DOT

**FIGURE 3:**
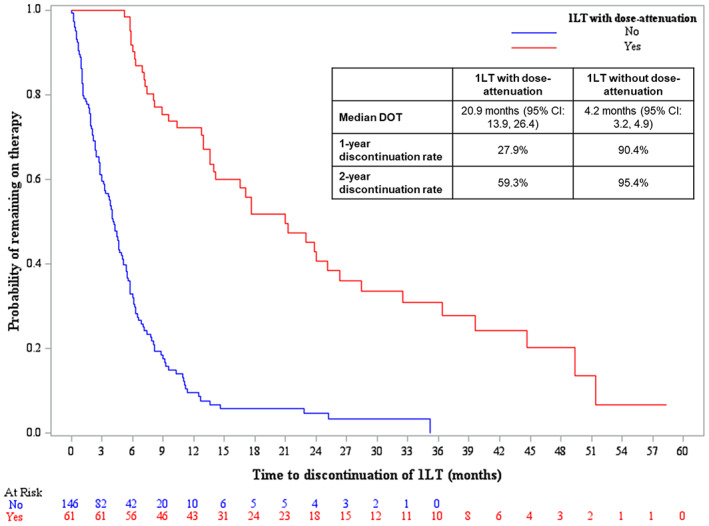
Kaplan–Meier curves of first‐line DOT and outcomes. 1LT, first‐line therapy; CI, confidence interval; DOT, duration of therapy.

The median DOT for 1LT was 20.9 months (95% CI: 13.9, 26.4) versus 4.2 months (95% CI: 3.2, 4.9) for patients receiving dose‐attenuation versus those who did not receive dose‐attenuation, respectively (*p* < 0.0001) (Figure 3). One‐ and 2‐year discontinuation rates in the dose‐attenuation cohort were 27.9% and 59.3%, respectively, and 90.4% and 95.4% in the cohort without dose‐attenuation, respectively. The unadjusted analyses of first‐line DOT for the top four induction regimens are provided in the Appendix S1.

### 3.4 Unadjusted and adjusted analysis of 1LT TTNT, PFS, and OS

**FIGURE 4:**
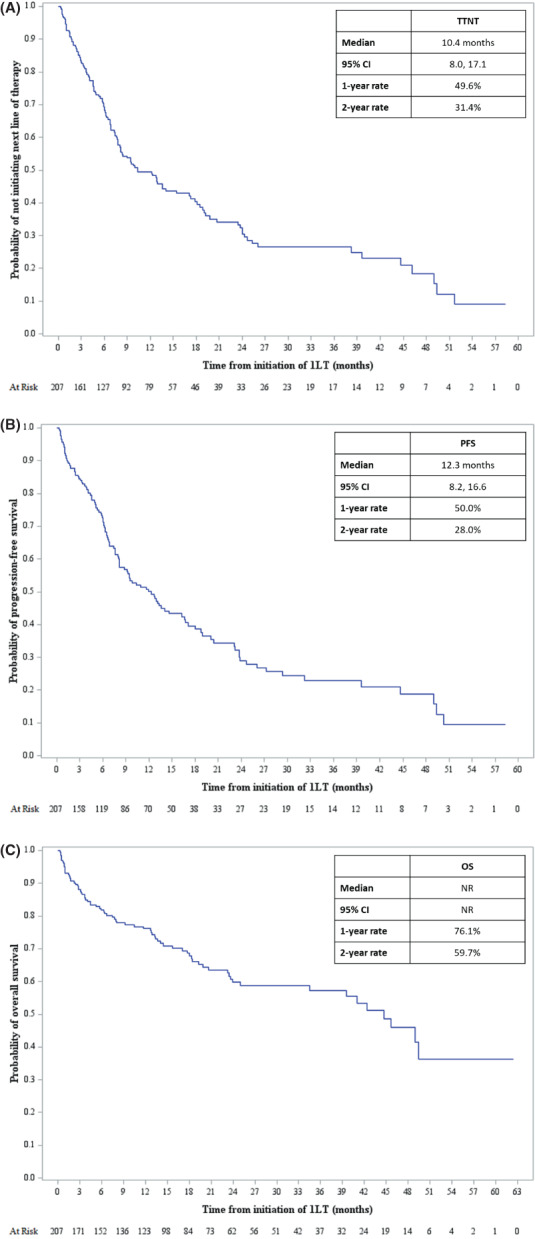
Kaplan–Meier curve of TTNT (A), PFS (B), and OS (C). CI, confidence interval; NR, not reported; OS, overall survival; PFS, progression‐free survival; TTNT, time‐to‐next therapy.

Unadjusted results, based on Kaplan–Meier analyses, indicated the 1LT median TTNT was 10.4 months (95% CI: 8.0, 17.1) and median PFS was 12.3 months (95% CI: 8.2, 16.6). Median OS was not reported due to extensive censoring (Figures 4A, 4B, 4C). The 1LT 1‐year rate of TTNT was 49.6%, PFS was 50.0%, and OS was 76.1%. The 2‐year 1LT TTNT, PFS, and OS rates were 31.4%, 28.0%, and 59.7%, respectively. Additional unadjusted analyses of outcomes (TTNT, PFS, and OS) stratified by the top four first‐line induction regimens (VRd, Vd, Rd, and VCd) are provided in the Appendix S1.

In the adjusted analyses, conducted using MSM to account for survival bias due to the time‐dependent nature of DOT, and patient and disease characteristics, TTNT, PFS, and OS at 2 years were improved with longer 1 L DOT (Table 3). Each additional month on 1LT was associated with reduced risk of initiating the next LOT or death at 2 years (OR: 0.76 [95% CI: 0.75, 0.78; *p* < 0.0001]). Similarly, each additional month on 1LT was associated with reduced risk of disease progression at 2 years (OR: 0.87 [95% CI: 0.86, 0.88; *p* < 0.0001]) and death at 2 years (OR: 0.72 [95% CI: 0.70, 0.74; *p* < 0.0001]).

**TABLE 3:** Adjusted OR for TTNT, progression, and OS using MSM

	OR	95% CI	% Risk reduction^a^	*p*‐value
TTNT	0.7603	0.7456, 0.7753	24	<0.001
PFS	0.8716	0.8606, 0.8827	13	<0.001
OS	0.7204	0.6992, 0.7423	28	<0.001

*Note*: Each additional month on 1LT was associated with reduced risk of initiating next LOT or death at 2 years (OR: 0.76 [95% CI: 0.75, 0.78]; *P* < 0.001), disease progression at 2 years (0.87 [95% CI: 0.86, 0.88]; *P* < 0.001), and death at 2 years (OR: 0.72 [95% CI: 0.70, 0.74]; *P* < 0.001).

Abbreviations: CI, confidence interval; LOT, line of therapy; MSM, marginal structural model; OR, odds ratio; OS, overall survival; PFS, progression‐free survival; TTNT, time to next treatment.

^a^ Percent risk reduction at 2 years.

## 4 DISCUSSION

This study suggests dose‐attenuation of 1LT may lead to significantly prolonged DOT as compared to patients who did not receive dose‐attenuation. Longer duration of 1LT was associated with reduced likelihood of disease progression, as measured by TTNT, PFS, and death at 2 years. This study, to our knowledge, is the first real‐world evidence analysis to evaluate dose‐attenuation of 1LT as a method to extend DOT in patients with NDMM without first‐line SCT. Our results are consistent with the hypotheses of the effects of extending DOT in relapsed/refractory multiple myeloma (RRMM) with dose‐attenuation.^26^, ^27^

Real‐world studies have evaluated dose‐attenuation in the treatment of patients with NDMM in 1LT but have focused on dose‐attenuation upfront in frail patients and the reported potential impact on outcomes. Tuchman et al studied patients with NDMM at high risk of therapy‐related toxicity due to their age or frailty treated with dose‐attenuated VCd, coined “VCd‐Lite” (n = 14). Results demonstrated an overall response rate of 64%, PFS of 24.2 months, and OS of 29.7 months. Patients discontinued therapy due to toxicity (14%), progression of disease (36%), and other reasons (29%).^28^ An additional study, RevLite, was a single‐arm, multicenter, phase 2 trial which evaluated lower starting doses of Rd in 150 patients with RRMM who were at risk of increased treatment‐related AEs. Seventy‐one percent of patients achieved at least a partial response (PR); median PFS was 8.9 months, and median OS was 30.5 months.^29^ While these studies evaluated upfront dose reductions in therapy, they did so for frail and at risk patients, and did not evaluate impact of extended DOT of 1LT with dose‐attenuated therapy following induction.

Similar to the studies above, a randomized, phase 3 trial evaluated maintenance therapy in a non‐SCT NDMM elderly population that was categorized as not “fit” by the International Myeloma Working Group frailty score. Patients were randomized to Rd until progression versus Rd induction followed by lenalidomide alone until progression. While there were no differences in PFS or OS between the continuous Rd versus Rd to lenalidomide maintenance therapy arms, the latter was associated with lower rates of AEs and dose reductions.^17^ Our study included predominantly elderly patients and results demonstrated a PFS and OS benefit with dose‐attenuation of 1LT patients with NDMM who did not undergo frontline SCT. Clinicians may consider utilizing dose‐attenuation approaches in non‐SCT NDMM populations to improve outcomes while potentially providing a more tolerable 1LT.

Other clinical trials have evaluated whether maintenance therapy versus observation or placebo‐controlled regimens could extend PFS. In the Myeloma XI trial, patients with SCT‐ineligible NDMM were stratified to lenalidomide maintenance versus observation with a reported PFS of 43 months (95% CI: 39, 48) and 35 months (95% CI: 31, 39), respectively (HR 0.72, 95% CI: 0.58, 0.88; *p* = 0.0016).^16^ No difference in OS was noted. Another study evaluated bortezomib‐melphalan‐prednisone‐thalidomide induction followed by bortezomib‐thalidomide maintenance (VMPT‐VT) versus bortezomib‐melphalan‐thalidomide (VMP) induction in patients with SCT‐ineligible NDMM. Patients were stratified to receive VMPT‐VT versus VMP induction only. Three‐year estimates of PFS among VMPT‐VT versus VMP were 56% and 41%, respectively (HR 0.67, 95% CI: 0.50, 0.90; *p* = 0.008).^30^ In the TOURMALINE‐MM4 trial, in patients with NDMM not undergoing SCT who achieved at least PR following 6–12 months of induction therapy, the median PFS was significantly better in those who received maintenance therapy (17.4 vs. 9.4 months, respectively; HR 0.66, 95% CI: 0.54, 0.80; *p* < 0.001).^31^, ^32^ These data indicate that prolongation of therapy improves PFS in NDMM which is in alignment with the first‐line DOT and treatment outcome findings in our study.

Another strategy utilized to extend DOT of 1LT is to employ in‐class transition (iCT) from a parenteral‐based induction to an all oral‐based regimen as demonstrated in the US MM‐6 trial.^33^ Utilization of an all‐oral MM regimen has been reported by patients as a more convenient and preferred treatment approach, has decreased healthcare resource utilization and indirect costs, and maintained high rates of therapy adherence.^33^, ^34^, ^35^, ^36^, ^37^ In addition to patient preference, transitioning patients to an all oral regimen may be beneficial in patients with decreased mobility or during times at which these immunocompromised patients may be at higher risk of contracting an infectious disease by reporting to healthcare settings—such as during the COVID‐19 pandemic. Patient exposure could be minimized with decreased interruptions to ongoing therapy without compromising efficacy.^19^, ^38^, ^39^, ^40^, ^41^ While our study did not examine iCT or focus on oral therapeutic options as a strategy to extend DOT, these may be reasonable approaches if deemed appropriate by healthcare providers for certain patients.

Frail and elderly patients with MM continue to remain an unmet medical need. Compared to SCT eligible patients, non‐SCT eligible patients may not receive multiple salvage therapies as they succumb to age and/or disease related comorbidities. Furthermore, DOT and PFS progressively diminish with each successive LOT.^12^ Therefore, given the short real world first‐line DOT in the non‐SCT eligible population, efforts should be made to attain deep responses *and* sustain remission with tolerable treatment approaches. Our real‐world findings align with the data from clinical trials which suggest that extending 1LT via maintenance therapy or a dose‐attenuation strategy extends PFS in patients with non‐SCT NDMM. Our findings demonstrated that dose‐attenuation could extend first‐line DOT and longer DOT may be associated with improved treatment outcomes of TTNT, PFS, and OS in these patients in routine community oncology clinic settings.

### 4.1 Limitations

This study has limitations that are typical of retrospective analyses. First, conceptually, there is no standard definition of dose‐attenuated therapy, limiting the ease of identifying these patients retrospectively; patients identified as receiving dose‐attenuated therapy in this study were verified by an expert MM oncologist with review of patient charts to ascertain the intention of drug or dose changes. Next, the sample size of patients with non‐SCT NDMM receiving dose‐attenuated therapy in 1LT was relatively small. Clinical characteristics such as cytogenetic abnormalities, ISS stage, and ECOG PS were impacted by missing data, limited or inconsistent testing and documentations across clinical sites. The reasons for not pursuing SCT were not collected during the medical chart review, however, other factors which determine a patient's transplant eligibility were collected and reported. Patients with the shortest DOT more commonly had an ECOG PS of 2 or greater, had high‐risk cytogenetics, and had higher CCI and modified frailty index scores of 2+, compared to patients with a longer DOT; however, these were adjusted for in the statistical analyses. Additionally, progression was determined based on physicians' documentation in the medical records; however, the criteria used to define progression by individual site physicians cannot be ascertained. Interestingly, our study observed a slightly shorter 1LT median TTNT compared to median PFS, which can be a reflection of variability in routine care practices or even chart documentation conventions; future studies should consider examining further both TTNT and PFS in other real‐world healthcare settings. Dosing was not captured in this study which would allow for cumulative dose intensity to be determined and may have served as a better correlate of outcomes as compared to DOT. In order to clinically enrich the patient data and verify accurate outcomes of interest not captured in structured data, a retrospective chart review was conducted on a random sample of patients from among NDMM without front‐line SCT patients that were identified in the ION database. Due to the resource‐intensive nature of chart review, this study included 300 randomly selected patients; thus, future research should consider other larger samples from routine care setting to confirm these findings. Finally, this research focused on community oncology clinics, so future studies should consider other oncology care settings.

## 5 CONCLUSIONS

Our current study indicates that the use of dose‐attenuated therapy is associated with longer DOT among patients with non‐SCT NDMM who remain on 1LT for 3 months or longer. Notably, an additional month on 1LT was associated with reduced likelihood of initiation of next LOT, disease progression, and death in this real‐world standard of care setting. This evidence suggests that dose‐attenuation of 1LT may lead to longer DOT, which may improve treatment outcomes and reduce the likelihood of death.

Supplementing clinical trial data, these real‐world findings highlight the feasibility of a positive impact on patients' treatment outcomes associated with longer first‐line DOT, which may be achieved with dose‐attenuated therapy in patients with NDMM without front‐line SCT. Further research is warranted to evaluate these findings in other patient populations and settings, particularly considering the dynamic and ever‐evolving treatment landscape of MM disease management.

## AUTHOR CONTRIBUTION

All authors participated in the development of the study concept, review of the results, and development of the manuscript. Eileen Farrelly, Augustina Ogbonnaya, and Sharanya Murty analyzed the data. All authors vouch for the accuracy and content of this manuscript and reviewed it prior to submission.

## FUNDING INFORMATION

Funding for this study was provided by Millennium Pharmaceuticals, Inc., a wholly owned subsidiary of Takeda Pharmaceutical Company Limited.

## CONFLICT OF INTEREST

Sikander Ailawadhi reports consulting or advisory fees from Amgen, Celgene, Janssen Biotech, Sanofi, GSK, Beigene and Takeda, research funding from Cellectar (Inst), Janssen Biotech (Inst), Pharmacyclics (Inst), BMS (inst), Xencor (inst), and Phosplatin Therapeutics (Inst). Dasha Cherepanov and Dorothy Romanus are employees of Millennium Pharmaceuticals, Inc., a wholly owned subsidiary of Takeda Pharmaceutical Company Limited. Augustina Ogbonnaya, Sharanya Murty, Bridgette Kanz Schroader, and Eileen Farrelly are employees of Xcenda, LLC. Ajai Chari reports grants and personal fees from Janssen, grants and personal fees from Celgene, grants and personal fees from Novartis Pharmaceuticals, grants and personal fees from Amgen, personal fees from Bristol Myers Squibb, grants from Pharmacyclics, personal fees from Karyopharm, personal fees from Sanofi, grants and personal fees from Seattle Genetics, personal fees from Oncopeptides, grants and personal fees from Millennium/Takeda, personal fees from Antengene, personal fees from GlaxoSmithKline, and personal fees from Secura Bio.

## ETHICS APPROVAL

The protocol for this study was reviewed and approved by the Advarra IRB (https://www.advarra.com/review‐services/institutional‐review‐board/).

## Supporting information


Appendix S1

Figure S1

Figure S2

Figure S3

Figure S4

Figure S5

## Data Availability

Data for this study were made available through a third‐party license from ION, a commercial data provider in the US; our analysis and results are reported in the main text and accompanying Appendix S1 . Further release of the dataset is not possible due to a data use agreement.
