# Supplementary material for: Duration of frontline therapy and impact on clinical outcomes in newly diagnosed multiple myeloma patients not receiving frontline stem cell transplant
Source: Cancer Med. 2022 Sep 24;12(3):3145–59. doi: 10.1002/cam4.5239 (PMC9939178; doi:10.1002/cam4.5239)
Supplement: Supplementary file 1 — Appendix S1 [file CAM4-12-3145-s004.docx]

**Appendix**

***Study Design Overview***

Below are definitions provided in the manuscript which correlate with Appendix Figure 1:

- **Study period:** January 1, 2012 to March 31, 2018.
- **Enrollment period:** July 1, 2012 to December 31, 2017.
- **Index diagnosis date:** First chronologically occurring multiple myeloma (MM) diagnosis during the enrollment period.
- **Index treatment date:** First chronologically occurring date of an MM chemotherapy during the enrollment period.
- **Baseline period:** 6-month period prior to index treatment date (but not including the index treatment date).
- **Follow-up period:** (including the index treatment date) was variable for all patients, and patients were followed longitudinally until death, loss to follow-up (that is, no additional data available prior to the end of study period), or end of study period.

***Definition of First-Line Regimen Categorization***

- Drug class
  - Immunomodulator drug (IMID)-based: Any monotherapy or combination that includes IMID (thalidomide, lenalidomide, or pomalidomide) but not a (proteasome inhibitor (PI) or alkylator or monoclonal antibody (mAB).
  - PI-based: Any monotherapy or combination that includes PI (bortezomib, carfilzomib, or ixazomib) but not IMID or alkylator or mAB.
  - Alkylator-based: Any combination or monotherapy that includes an alkylator (melphalan, BCNU, cyclophosphamide) but no IMID or PI or mAB.
  - mAB-based: Any monotherapy or combination that includes mAB (daratumumab, elotuzumab) but not IMID or alkylator or PI.
  - PI/IMID-based: Any combination that includes PI (bortezomib, carfilzomib, or ixazomib) and an IMID (thalidomide, lenalidomide, pomalidomide) but not alkylator or mAB.
  - PI/alkylator-based: Any combination that includes PI (bortezomib, carfilzomib, ixazomib) and alkylator (melphalan, BCNU, cyclophosphamide) but not IMID or mAB.
  - PI/IMID/alkylator-based: Any combination that includes PI (bortezomib, carfilzomib, ixazomib), an IMID (thalidomide, lenalidomide, pomalidomide), and alkylator (melphalan, BCNU, cyclophosphamide) but not mAB.
  - IMID/alkylator-based: Any combination that includes IMID (thalidomide, lenalidomide, pomalidomide) and alkylator (melphalan, BCNU, cyclophosphamide) but not PI or mAB.
  - mAB/IMID-based: Any combination that includes mAB (daratumumab or elotuzumab) and an IMID (thalidomide, lenalidomide, pomalidomide) but not alkylator or not PI.
  - mAB/PI-based: Any combination that includes mAB (daratumumab or elotuzumab) and PI (bortezomib, carfilzomib, or ixazomib) but not IMID or not alkylator.
  - mAB/alkylator-based: Any combination that includes mAB (daratumumab or elotuzumab) and alkylator (melphalan, BCNU, cyclophosphamide) but not PI or not IMID.
  - mAB/IMID/PI-based: Any combination that includes mAB (daratumumab or elotuzumab) and an IMID (thalidomide, lenalidomide, pomalidomide) and PI (bortezomib, carfilzomib, or ixazomib) but not alkylator.
- Drug count
  - Doublet, ≤2 drugs
  - Triplet, ≥3 drugs

***Definitions of Demographics***

- Age at initiation of 1LT (<65, 65-74, and ≥75)
- Gender (male and female)
- Race (African American, Caucasian, other/unknown)
- Ethnicity (Hispanic, non-Hispanic, unknown)

***Definitions of Clinical Characteristics***

- Year of MM diagnosis (2012-2017)
- Year of index treatment date (ie, start of induction therapy; 2012-2017)
- SCT-eligible (yes, no, unknown) was defined based on data captured from review of the progress notes.
- Eastern Cooperative Oncology Group (ECOG) performance status (PS) (0, 1, 2, 3, 4, unknown) was defined based on data captured from review of the progress notes.
- International staging system (ISS) stage (stage I, II, III, and unknown/not reported) was defined based on data captured from review of the progress notes.
- Immunoglobulin class (IgA, IgD, IgE, IgG, IgM, light chain only, biclonal, unknown, other) was defined based on data captured from review of the progress notes.
- Cytogenetic risk tested (yes [high/normal per below] vs no [unknown/not reported]) was defined based on data captured from review of the progress notes.
- Cytogenetic risk level was defined based on data captured from review of the progress notes and categorized as:
  - High: presence of del[17p], t[4;14], t[14;16], and/or 1q21 gain
  - Normal: no presence of above
  - Unknown/not reported
- Quan’s adaptation of the CCI^22^ (categorized: 0, 1, 2+), the individual comorbidities will be defined based on the presence of at least one diagnosis code in the structured data field or record in the progress notes:
  - Myocardial infarction
  - Cerebrovascular disease
  - Congestive heart failure
  - Chronic pulmonary disease
  - Peptic ulcer disease
  - Peripheral vascular disease
  - Mild liver disease
  - Moderate to severe liver disease
  - Connective tissue disease
  - Diabetes
  - Diabetes with end organ damage
  - Dementia
  - Hemiplegia
  - Moderate to severe renal disease
  - Any malignancy (except MM)
  - Metastatic solid tumor
  - HIV/AIDS
- Select comorbidities (yes vs no) was defined based on the presence of at least one diagnosis code in the structured data field or record in the progress notes:
  - Diabetes
  - Thromboembolic disease
  - Peripheral neuropathy
  - Cardiovascular disease
  - Prior non-MM cancer
- Modified frailty index (0=fit, 1=intermediate, 2+=frail) was defined as an additive score of the following criteria^8^:
  - Age, years
    - ≤75: Score=0
    - 76-80: Score=1
    - >80: Score=2
  - CCI score
    - ≤1: Score=0
    - ≥2: Score=1
- CRAB symptoms and the component diagnoses (yes vs no) were identified by the presence of at least one diagnosis code or lab value or documentation in the progress notes as defined below:
  - Bone disease, proxied by diagnosis codes for fracture, radiation, bone-directed surgery, spinal cord compression.^24^
  - Renal failure was defined using specific diagnosis codes, or laboratory test results indicating serum creatinine level >2 mg/dL or creatinine clearance <40 mL/min.^23^
    - Hypercalcemia was defined using specific diagnosis codes, or laboratory test results indicating corrected calcium level >11.0 mg/dL or >1 mg/dL higher than upper limit normal (normal range: 8.9–10.1 mg/dL).^23^
      - *Corrected calcium level was calculated as* ***0.8*** ** (****4*** *– albumin result) + calcium result*.^42^
  - Anemia was defined using specific diagnosis codes, or laboratory test results indicating hemoglobin level <10 g/dL or >2 g/dL lower than lower limit normal (normal range: 13.5–17.5 g/dL for men; 12.0–15.5 g/dL for women).^23^

**Definition of Conceptual LOT**

Conceptual Definition LOT^11,20,21^

The following criteria were used to identify a LOT:

1. A new line of therapy is considered to start when a planned course of therapy is modified to include other treatment agents (alone or in combination; eg, switch in at least one agent or add-on of an agent, other than steroids) as a result of progressive disease, relapse, sub-optimal response/lack of response, or toxicity.
2. Induction therapy followed by dose-attenuation (provided there is no intervening progressive disease) are considered to be a single LOT.

**Definition of Conceptual Dose-Attenuation**

Conceptual definition of dose-attenuation:

In the first-line setting:

In general, for observational studies, the following is a conceptual definition of dose-attenuated therapy in the first-line setting:

1. Change in polypharmacy (eg, triplet/doublet) to doublet- or mono-therapy, doublet-therapy accompanied by a dose reduction and/or a sustained decrease in frequency of administration – from an induction/consolidation regimen to a subset of the initial regimen, or to a single agent in non-transplant 1LT.
   - 1. A decrease of frequency/dose followed by resumption of original frequency/dose will not be considered maintenance (to account for real-world practice when patients may not be seen exactly on schedule).
2. Switch within the same mechanism of action from a PI-containing/IMID-containing induction/consolidation regimen in non-transplant/transplant 1LT to single-agent PI/IMID therapy, respectively (eg, bortezomib-containing 🡪 ixazomib, lenalidomide-containing 🡪 pomalidomide), could be considered maintenance under the following scenarios in a sequential order:

Utilizing measures of disease (if robust longitudinal data are available) at the time of 1 to 2 above:

- - 1. Serum M-protein (IgG Kappa): plateaus within 0.2 g/dL for 2 to 3 cycles or is undetectable.
    2. Free light chains (FLC) (among patients with oligo- or non-secretory MM; normal is ~20 [3.3–19.4 mg/L and 5.7–26.6 mg/L for the κ and λ chains, respectively]): involved FLC ratio plateaus; ie, ≥50% reduction in involved light chain (Kappa or Lambda) for 2 to 3 cycles from highest level, or current involved FLC ratio is normal.

1. If laboratory measures are not available in source data, use physician’s documentation of depth of response; ie, a stringent complete response/complete response (CR), or very good partial response (VGPR)/partial response (PR) for 2 to 3 cycles.

**Marginal structural models (MSM)**

To evaluate the impact of 1L DOT on TTNT, PFS, and OS, MSM were used.^25^ MSM is a multi-step estimation statistical model used to assess the impact of an exposure variable (eg, first-line DOT) that change over time on outcomes while controlling for confounding variables (eg, patient characteristics) that change over time. In our study, the MSM was utilized to account for the time-varying nature of first-line DOT and time-varying nature of confounders using inverse probability of treatment weights. The inverse probability of treatment weights were derived using propensity scores generated from logistic regression models.

The first propensity score logistic regression model created a propensity score for being on 1LT at each month during follow-up. The second set of logistic models estimated the probability of the following outcomes for each month from the start of 1LT for each month from the start of 1LT: 1) OS: being in the study; 2) PFS: being in the study progression-free; and 3) TTNT: being in the study and not on new therapy at each month of follow-up. The propensity scores derived from the models described above were used to generate weights at each month of follow-up. The weights were generated by multiplying the propensity scores from the logistic regression models which assessed the probability of continuing 1LT and the propensity scores from the models which assessed the probability of being in the study. These weights generated from the propensity score models described above estimated bias attributable to the extent to which patients with certain characteristics are under-represented or over-represented in the study sample. Outliers in the final weights were not trimmed.

Propensity scores and MSMs included both static covariates and time-varying covariates. Static covariates were age, gender, race, region, year of index treatment start, ISS stage, immunoglobulin class, cytogenetic risk, frailty (propensity score model only), CCI, and time from diagnosis to initiation of 1LT, while the time-varying covariates were ECOG PS, diabetes, thromboembolic disease, peripheral neuropathy, non-MM cancer, and cardiovascular disease, with data updated monthly.

Outcomes of TTNT, PFS, and OS were dichotomized according to whether each event had occurred at each month of follow-up. Once an event occurred, the event was positive for the remainder of the time periods. Logistic regression models for the dichotomized outcomes of TTNT, PFS, and OS were fit over all months, through 2 years of follow-up, incorporating the corresponding time-varying and non-time varying propensity weights and time varying covariates. This model estimated the propensity-weighted and covariate-adjusted average odds of having the outcome of interest for 1 additional month since the time of initiation of 1LT at 2 years of 1LT treatment.

In the final MSM, the outcomes (TTNT/PFS/OS, as indicator variables) were truncated at 2 years because of extensive censoring and small number of patients remaining following 2 years of follow-up (n=33, 27, and 62 for TTNT, PFS, and OS, respectively). Results from the MSM were summarized as multivariable adjusted ORs (and associated 95% CI) for having the event for the given outcome for each additional month of continuous therapy among those who continued vs those who discontinued 1LT.

**Appendix Tables**

**Appendix Table 1. Unadjusted analysis: Duration of 1LT by top four first-line induction regimens in patients with NDMM without front-line SCT**

| **DOT** | **All patients** | **Top four first-line induction regimens** | | | |
| --- | --- | --- | --- | --- | --- |
|  |  | **VRd** | **Vd** | **Rd** | **VCd** |
|  | **N=207** | **N=63** | **N=56** | **N=38** | **N=34** |
| Median duration of 1LT, months (95% CI) | 6.0 (5.4, 7.1) | 6.5 (5.1, 8.1) | 5.7 (4.6, 7.4) | 6.1 (3.7, 13.0) | 5.8 (2.0, 9.3) |
| 1-year rate of remaining on therapy | 29.8% | 33.4% | 20.7% | 37.1% | 29.6% |
| 2-year rate of remaining on therapy | 16.3% | 22.9% | 11.0% | 22.5% | 12.7% |

Key: 1LT – first-line therapy; CI – confidence interval; DOT – duration of therapy; IQR – interquartile range; NDMM – newly diagnosed multiple myeloma; Rd – lenalidomide, dexamethasone; SCT – stem cell transplant; SD – standard deviation; VCd – bortezomib, cyclophosphamide, dexamethasone; Vd – bortezomib, dexamethasone; VRd – bortezomib, lenalidomide, dexamethasone.

**Appendix Table 2. Unadjusted analysis: TTNT by top four first-line induction regimens in patients with NDMM without front-line SCT**

| **TTNT** | **All patients** | **Top four first-line induction regimens** | | | |
| --- | --- | --- | --- | --- | --- |
|  |  | **VRd** | **Vd** | **Rd** | **VCd** |
|  | **N=207** | **N=63** | **N=56** | **N=38** | **N=34** |
| Median TTNT, months (95% CI) | 10.4 (8.0, 17.1) | 13.6 (7.4, 38.2) | 8.0 (6.7, 12.8) | 7.8 (4.5, 15.4) | 18.4 (6.0, 23.5) |
| 1-year TTNT rate | 49.6% | 51.6% | 40.8% | 44.3% | 55.3% |
| 2-year TTNT rate | 31.4% | 43.9% | 19.1% | 33.0% | 25.4% |

Key: CI – confidence interval; NDMM – newly diagnosed multiple myeloma; Rd – lenalidomide, dexamethasone; SCT – stem cell transplant; SD – standard deviation; TTNT – time to next therapy; VCd – bortezomib, cyclophosphamide, dexamethasone; Vd – bortezomib, dexamethasone; VRd – bortezomib, lenalidomide, dexamethasone.

**Appendix Table 3.** **Unadjusted analysis: PFS by top four first-line induction regimens in patients with NDMM without front-line SCT**

| **PFS** | **All patients** | **Top four first-line induction regimens** | | | |
| --- | --- | --- | --- | --- | --- |
|  |  | **VRd** | **Vd** | **Rd** | **VCd** |
|  | **N=207** | **N=63** | **N=56** | **N=38** | **N=34** |
| Median PFS, months (95% CI) | 12.3 (8.2, 16.6) | 12.7 (7.6, 23.1) | 9.5 (6.5, 16.6) | 13.6 (5.1, NE) | 11.6 (6.5, 19.0) |
| 1-year PFS rate | 50.0% | 51.0% | 46.9% | 52.9% | 45.4% |
| 2-year PFS rate | 28.0% | 31.4% | 24.6% | 43.2% | 20.2% |

Key: CI – confidence interval; NDMM – newly diagnosed multiple myeloma; NE – not estimable; PFS – progression-free survival; Rd – lenalidomide, dexamethasone; SCT – stem cell transplant; SD – standard deviation; VCd – bortezomib, cyclophosphamide, dexamethasone; Vd – bortezomib, dexamethasone; VRd – bortezomib, lenalidomide, dexamethasone.

**Appendix Table 4.** **Unadjusted analysis: OS by top four first-line induction regimens in patients with NDMM without front-line SCT**

| **OS** | **All patients** | **Top four first-line induction regimen** | | | |
| --- | --- | --- | --- | --- | --- |
|  |  | **VRd** | **Vd** | **Rd** | **VCd** |
|  | **N=207** | **N=63** | **N=56** | **N=38** | **N=34** |
| Median OS, months (95% CI) | 44.7 (34.5, NE) | 42.4 (23.7, NE) | 24.0 (15.7, NE) | NR (NE, 19.9) | 45.6 (23.3, NE) |
| 1-year OS rate | 76.1% | 72.0% | 77.7% | 77.0% | 78.0% |
| 2-year OS rate | 59.7% | 64.3% | 48.0% | 67.4% | 66.7% |

Key: CI – confidence interval; NDMM – newly diagnosed multiple myeloma; NE – not estimable; NR – not reached; OS – overall survival; Rd – lenalidomide, dexamethasone; SCT – stem cell transplant; SD – standard deviation; VCd – bortezomib, cyclophosphamide, dexamethasone; Vd – bortezomib, dexamethasone; VRd – bortezomib, lenalidomide, dexamethasone.

**Appendix Figure Legends**

**Appendix Figure 1. Study period schematic**

^†^ Diagnosis date could occur any time at or before the index treatment date.

**Appendix Figure 2. Kaplan-Meier estimate of duration of first-line therapy by top four first-line induction regimens in patients with NDMM without front-line SCT**

Key: 1LT – first-line therapy; NDMM – newly diagnosed multiple myeloma; SCT – stem cell transplant.

**Appendix Figure 3. Kaplan-Meier estimate of TTNT by top four first-line induction regimens in patients with NDMM without front-line SCT**

Key: NDMM – newly diagnosed multiple myeloma; SCT – stem cell transplant; TTNT – time to next therapy.

**Appendix Figure 4. Kaplan-Meier estimate of PFS by top four first-line induction regimens in patients with NDMM without front-line SCT**

Key: FLT – first-line therapy; NDMM – newly diagnosed multiple myeloma; PFS – progression-free survival; SCT – stem cell transplant.

**Appendix Figure 5. Kaplan-Meier estimate of OS by top four first-line induction regimens in patients with NDMM without front-line SCT**

Key: FLT – first-line therapy; NDMM – newly diagnosed multiple myeloma; OS – overall survival; SCT – stem cell transplant.

**APPENDIX REFERENCES**

Carroll MF, Schade DS. A practical approach to hypercalcemia. *Am Fam Physician*. 2003;67(9):1959-1966.

Chari A, Richardson PG, Romanus D, et al. Real-world outcomes and factors impacting treatment choice in relapse and/or refractory multiple myeloma (RRMM): a comparison of VRd, KRd, and IRd. *Expert Rev Hematology*. 2020a; 13(4):421-433. doi: 10.1080/17474086.2020.1729734.

Nash Smyth E, Conti I, Wooldridge JE, et al. Frequency of skeletal-related events and associated healthcare resource use and costs in US patients with multiple myeloma. *J Med Econ*. 2016;19:5:477-486.

Referenced with permission from the NCCN Clinical Practice Guidelines in Oncology (NCCN Guidelines^®^) for Multiple Myeloma Version 4.2022. © National Comprehensive Cancer Network, Inc. 2021. All rights reserved. Accessed December 27, 2021. To view the most recent and complete version of the guideline, go online to NCCN.org. NCCN makes no warranties of any kind whatsoever regarding their content, use or application and disclaims any responsibility for their application or use in any way.

Palumbo A, Bringhen S, Mateos MV, et al. Geriatric assessment predicts survival and toxicities in elderly myeloma patients: an International Myeloma Working Group report. *Blood*. 2015a;125:2068-2074.

Quan H, Sundararajan V, Halfon P, et al. Coding algorithm for defining comorbidities in ICD-9-CM and ICD-10 administrative data. *Med Care*. 2005;43(11):1130-1139.

Rajkumar SV, Harousseau JL, Durie B, et al. Consensus recommendations for the uniform reporting of clinical trials: report of the international myeloma workshop consensus panel 1. *Blood*. 2011;117(18):4691-4695.

Rajkumar SV, Richardson P, San Miguel JF. Guidelines for determination of the number of prior lines of therapy in multiple myeloma. *Blood.* 2015;126(7):921-922.
