# Supplementary figures and images for: Duration of frontline therapy and impact on clinical outcomes in newly diagnosed multiple myeloma patients not receiving frontline stem cell transplant
Source: Cancer Med. 2022 Sep 24;12(3):3145–59. doi: 10.1002/cam4.5239 (PMC9939178; doi:10.1002/cam4.5239)

## Slide 1
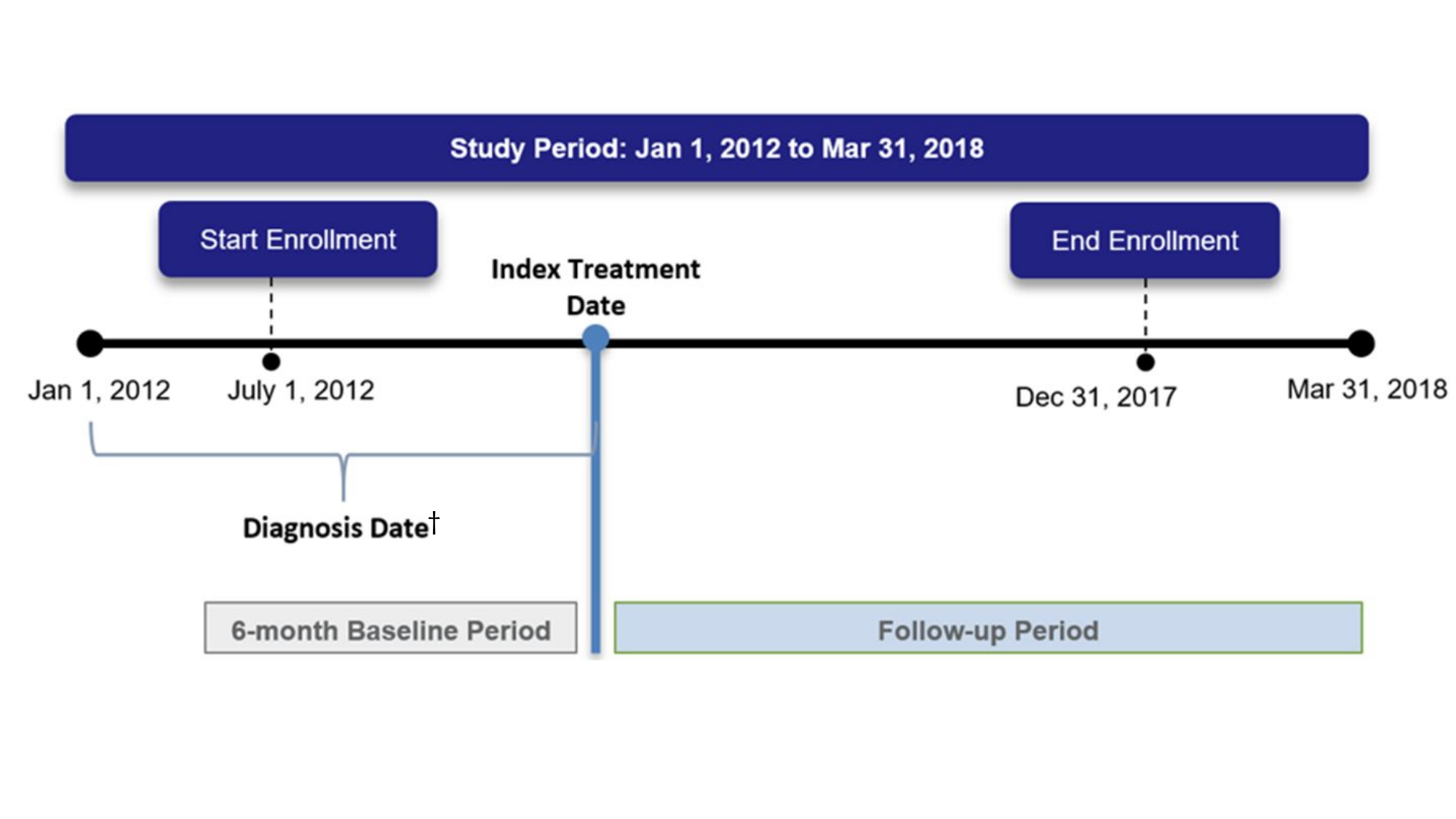

†

Supplement: Supplementary file 2 — Figure S1 [file CAM4-12-3145-s001.pptx]

## Slide 1
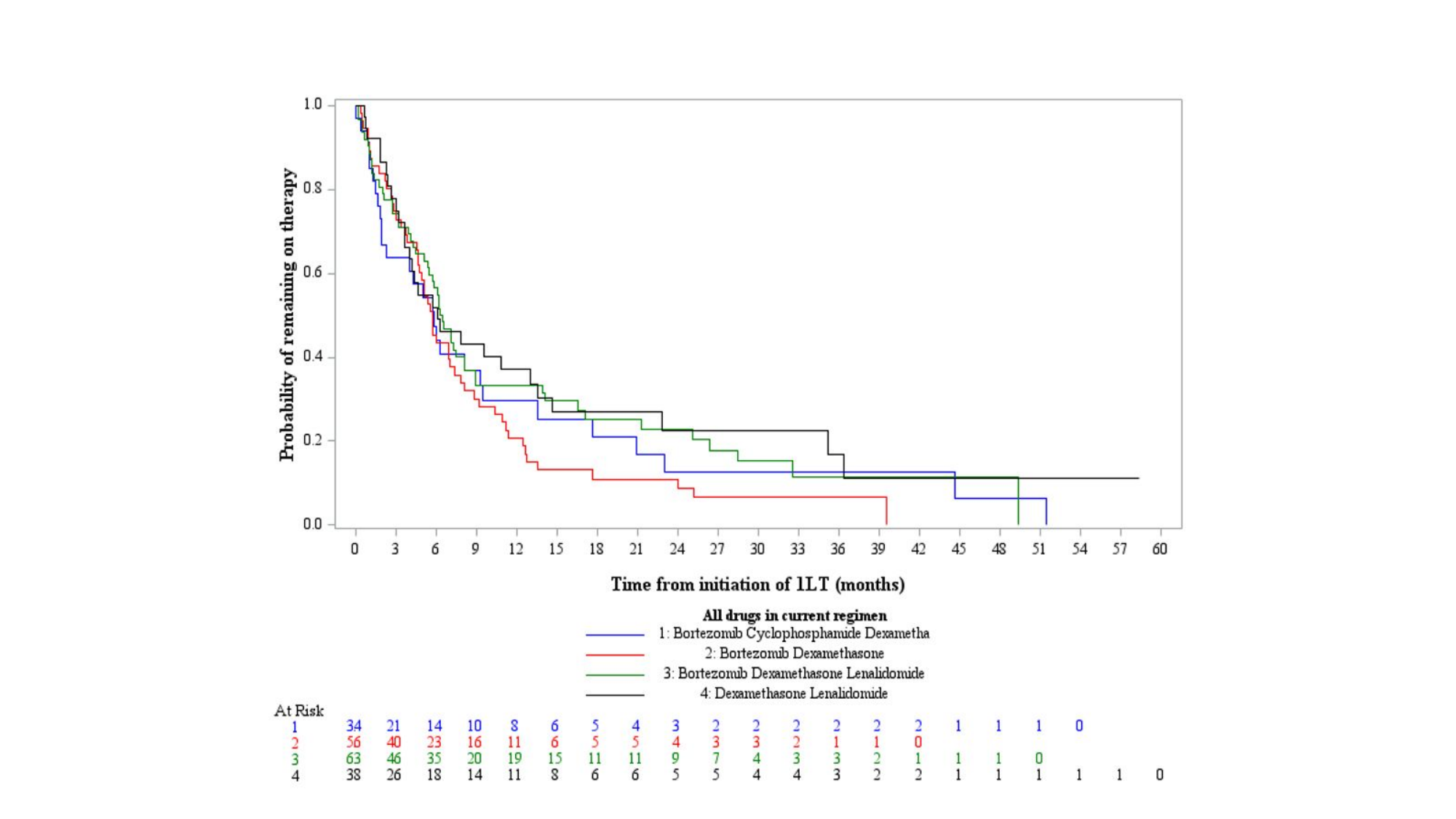

Supplement: Supplementary file 3 — Figure S2 [file CAM4-12-3145-s002.pptx]

## Slide 1
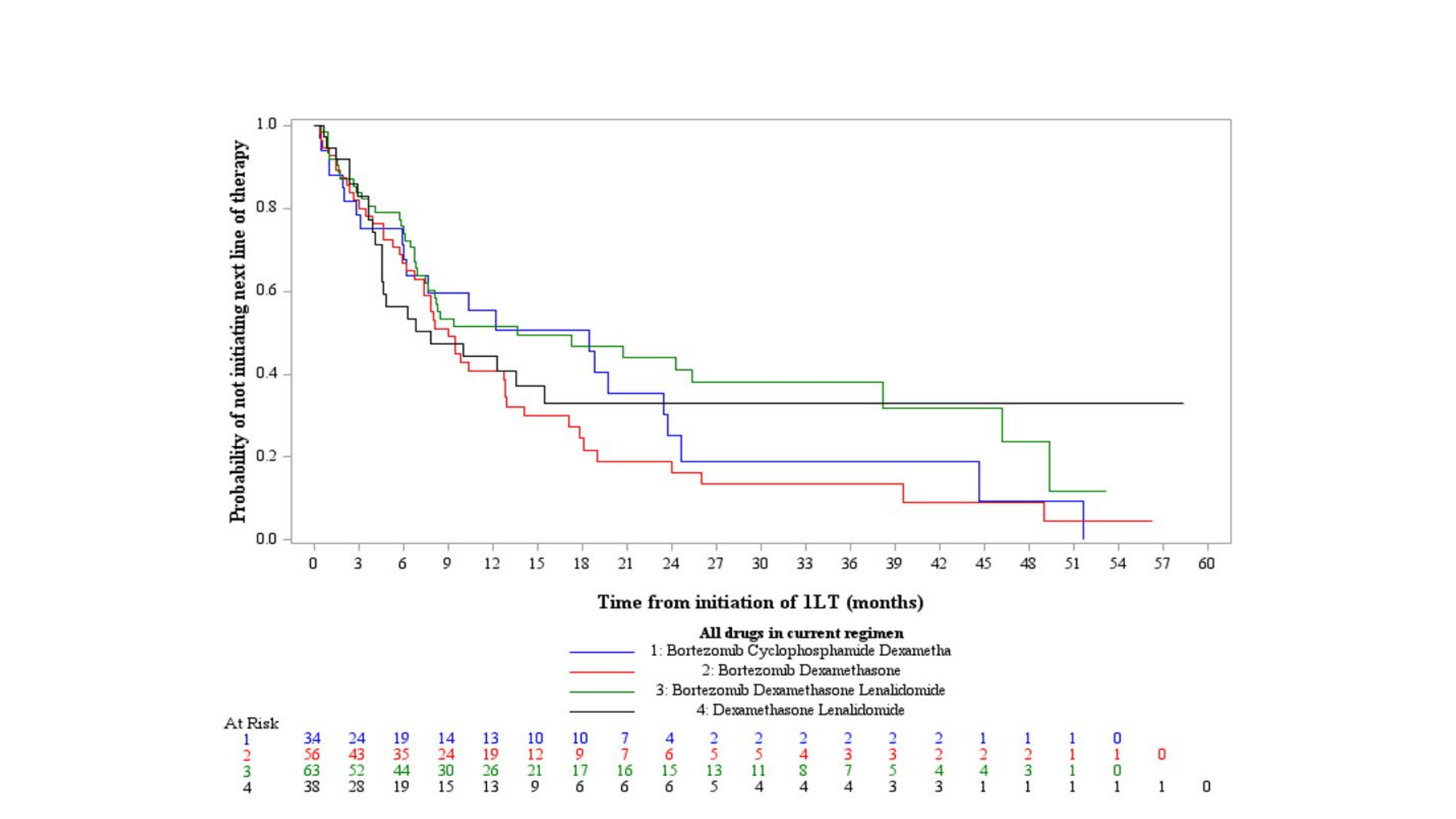

Supplement: Supplementary file 4 — Figure S3 [file CAM4-12-3145-s003.pptx]

## Slide 1
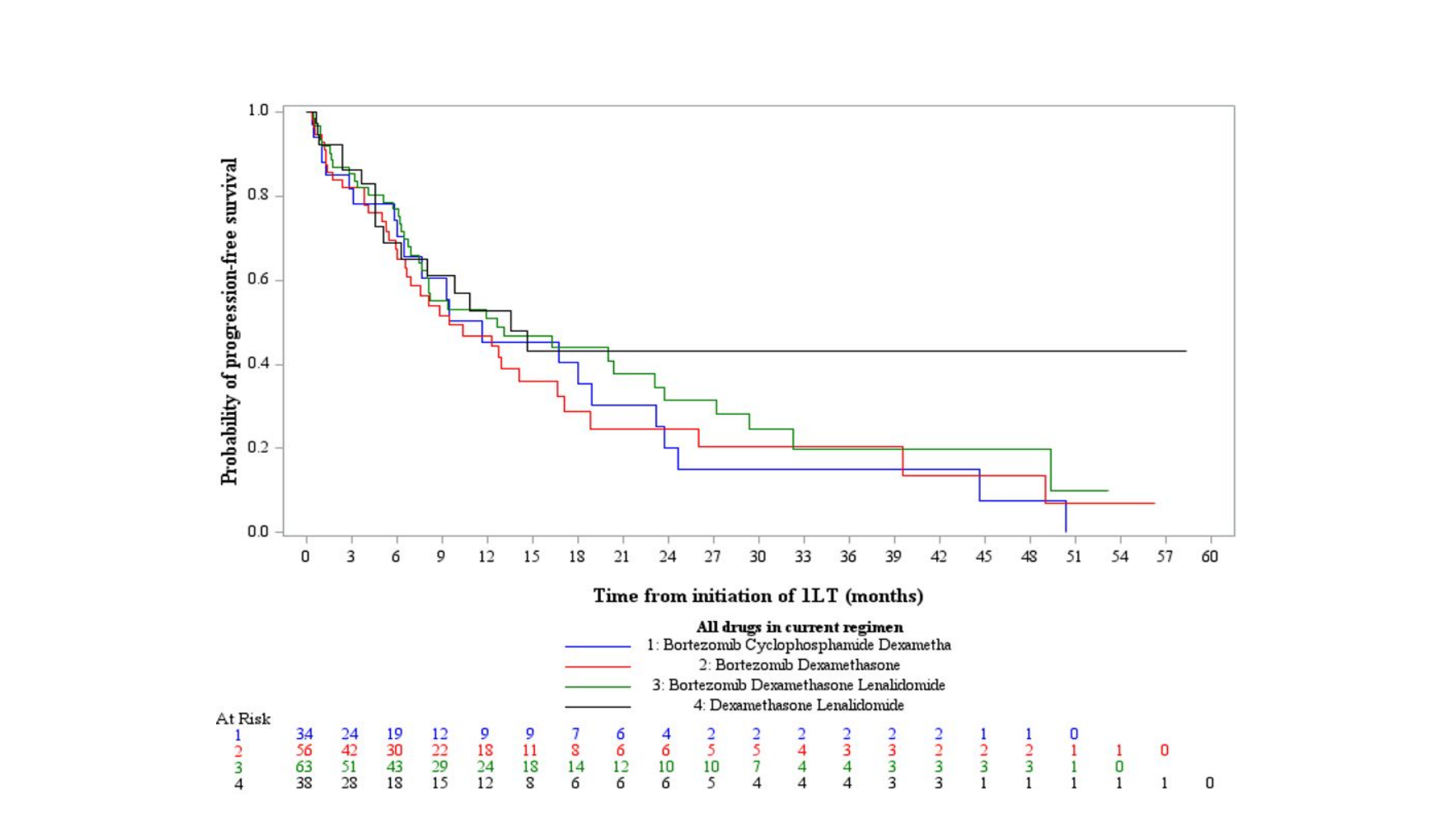

Supplement: Supplementary file 5 — Figure S4 [file CAM4-12-3145-s005.pptx]

## Slide 1
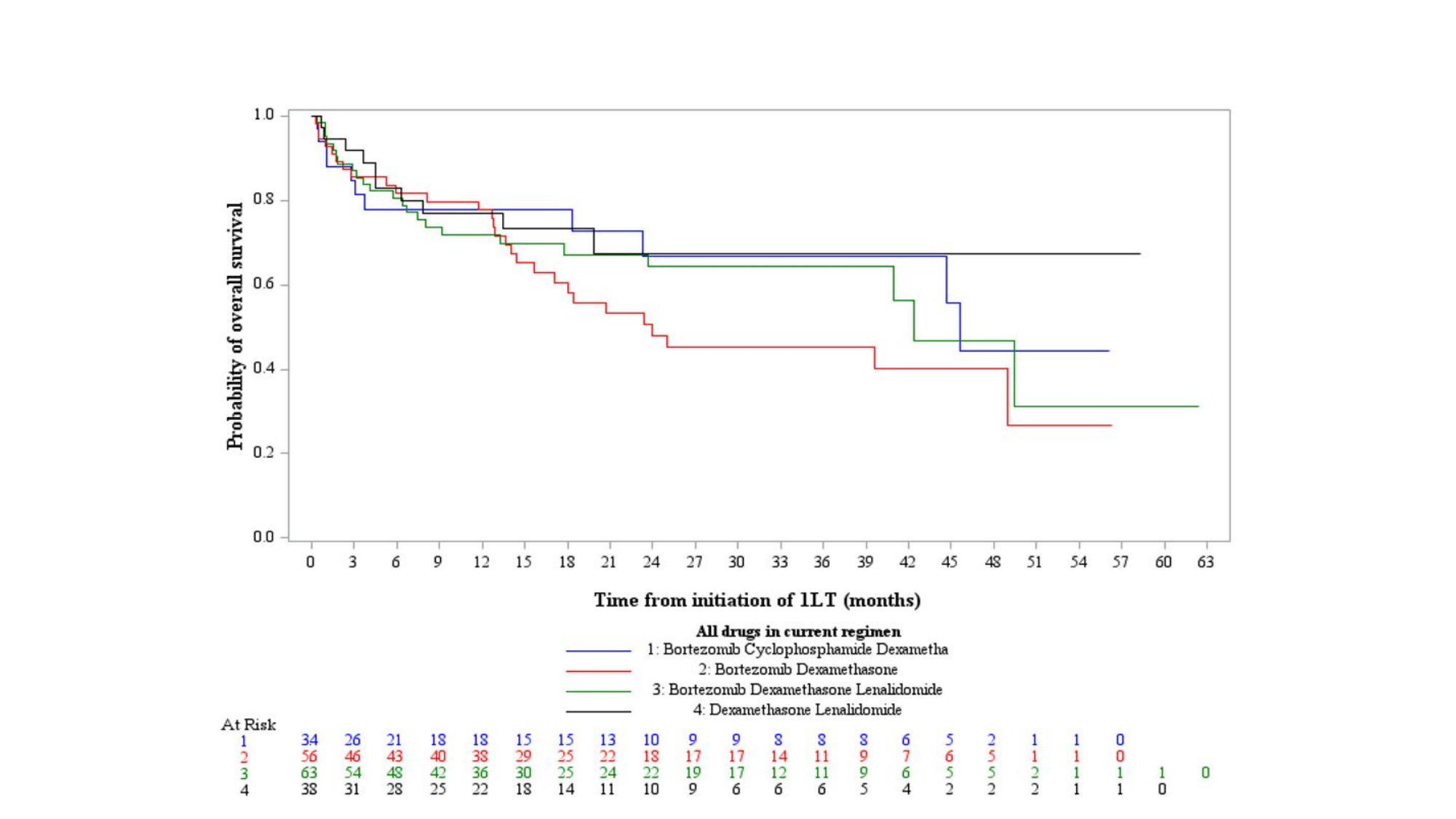

Supplement: Supplementary file 6 — Figure S5 [file CAM4-12-3145-s006.pptx]
